# Zoster-associated limb paralysis mimicking acute stroke: a case report

**DOI:** 10.1186/s13256-021-02971-7

**Published:** 2021-07-30

**Authors:** Chamara Dalugama, Ruwanthi Jayasinghe, Nimanthi Rathnayaka, Arjuna Medagama

**Affiliations:** 1 Department of Medicine, University of Peradeniya, Kandy, Sri Lanka; 2 University Medical Unit, Teaching Hospital, Peradeniya, Kandy, Sri Lanka; 3 University Medical Unit, Teaching Hospital, Peradeniya, Kandy, Sri Lanka; 4 Department of Medicine, University of Peradeniya, Kandy, Sri Lanka

**Keywords:** Varicella-zoster, Motor neuropathy, Plexopathy, Stroke

## Abstract

**Background:**

Varicella zoster virus is a Deoxyribonucleic acid (DNA) virus exclusively affecting humans. Reactivation of varicella zoster virus causes herpes zoster with vesicular eruptions in a restricted dermatomal distribution. Peripheral motor neuropathy is a very rare complication of varicella zoster virus.

**Case presentation:**

A 57-year-old previously well Sri Lankan female presented with acute onset painful weakness of the left upper limb with a preceding history of a febrile illness. Subsequently she developed vesicular eruptions in the dermatomal distribution of cervical 5, 6, and 7. Electromyography was suggestive of acute denervation of cervical 5, 6, and 7 myotomes. Diagnosis of zoster-associated brachial plexopathy was made, and the patient was treated with acyclovir, steroids, and analgesics. She made a good recovery.

**Conclusion:**

Brachial plexus neuritis due to varicella zoster infection should be considered in an acute monoparesis of a limb as it is a treatable and reversible condition

## Introduction

Varicella zoster virus (VZV) is a Deoxyribonucleic acid (DNA) virus in the Herpesviridae family [1]. It is known to affect humans exclusively. Primary infection with VZV causes vesicular lesions on erythematous base at different stages of development mainly concentrated on trunk and face [2]. Reactivation of latent VZV that gained access to sensory ganglia during varicella causes herpes zoster that characteristically causes unilateral vesicular eruptions in a restricted dermatomal distribution [3]. However, VZV is known to present with uncommon neurological manifestations such as aseptic meningitis, encephalitis, myelitis, and, rarely, peripheral motor neuropathy [4]. Sepsis, autoimmune disease, and radiation can often lead to immunosuppression, which can often worsen the prognosis of herpes zoster [5, 6]

Peripheral motor neuropathy due to zoster is a very uncommon complication due to varicella zoster. It can result in acute limb paralysis mimicking a stroke. We report the case of a previously healthy female presented with acute monoparesis of the upper limb with subsequent development of the vesicular rash involving cervical 5, 6, and 7 dermatomes.

## Case presentation

A previously healthy 57-year-old Sri Lankan lady from Kandy, Sri Lanka presented with weakness of the left upper limb that was noticed on awakening form sleep 2 days prior to admission. Weakness was steadily progressing, and patient was unable to move the left upper limb on admission. She had generalized malaise since 3 days preceding the weakness with low-grade on-and-off fever with pain radiating to the left upper limb. The patient denied any weakness of the bilateral lower limbs or bladder and bowel incontinence. She has not noticed in speech difficulty, swallowing difficulty, or weakness of the face. She did not have any headache, double vision, vomiting, or visual changes with the onset of symptoms. There was no history of neck pain or trauma preceding the weakness.

**Fig. 1:**
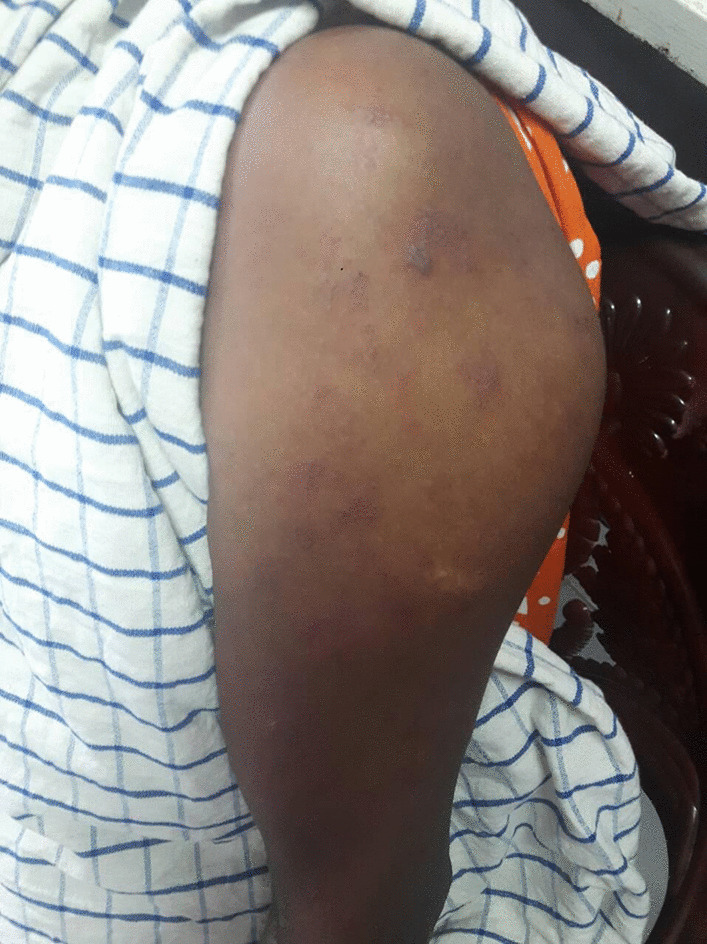
Vesicular rash in erythematous base suggestive of varicella zoster

**Fig. 2:**
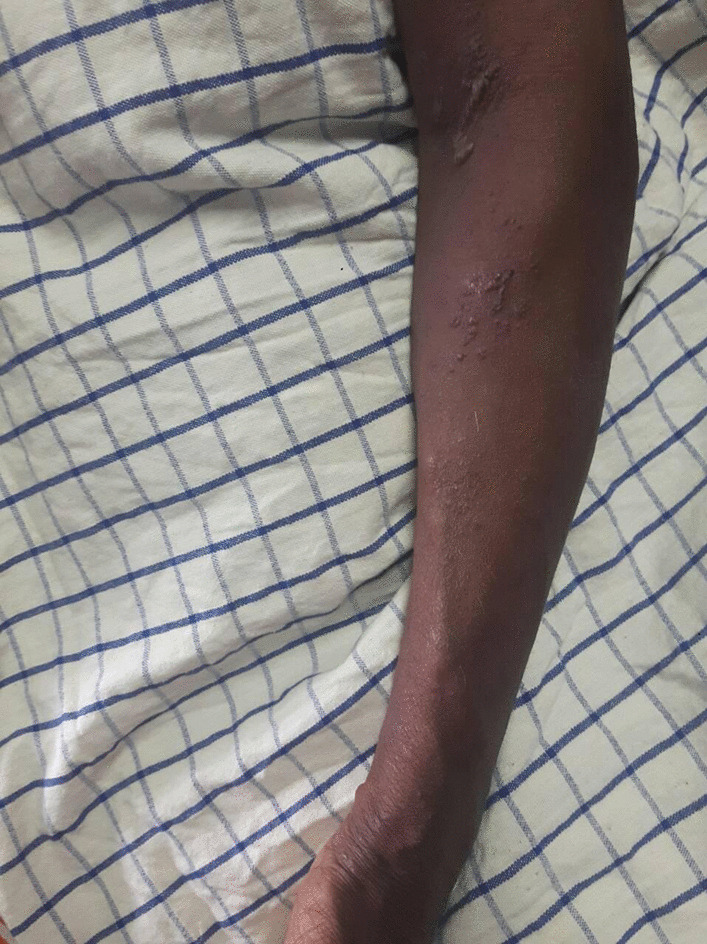
Vesicular rash in erythematous base suggestive of varicella zoster

On admission, the patient was in distress due to left upper limb pain. She was oriented in time, place, and person. She was not pale. Pulse rate was 88 beats per minute, which was regular. Her blood pressure was 130/90 mmHg with normal precordium examination. Respiratory system was unremarkable. Neurological examination revealed flaccid paralysis of the left upper limb with proximal weakness (0/5) more than the distal (2/5). Her left upper limb reflexes were diminished. She had hyperesthesia in the left upper limb during sensory examination without any objective sensory loss. The rest of the neurological examination was normal, including cranial nerves and lower limbs. Non-contrast computed tomography (CT) of the brain showed no territorial infarct or acute bleed to explain the monoparesis. On day 2 of admission, the patient complained of a painful rash involving the left upper limb. On examination, she had a vesicular rash on an erythematous base suggestive of varicella zoster (Figs. 1 and 2)

Her white cell count was 8.7 × 10^6^/dL (neutrophils 70%) with a platelet count of 234 × 10^3^/dL and hemoglobin of 12.5 g/dL. C-reactive protein was less than 6 units, and erythrocyte sedimentation rate was 12 mm in the first hour. Her transaminases were normal with normal renal functions. Cervical spine X-ray revealed a straight spine suggestive of muscle spasm. Nerve conduction study showed normal median nerve conduction and electromyography (EMG) suggestive of active denervation in the left cervical 5, 6, and 7 myotomes suggestive of a lesion involving brachial plexus. Her cerebrospinal fluid (CSF) analysis revealed a white count of 330 per high-power field with 80% lymphocytes and protein of 130 g/L. Her CSF sugar was 3.6 mmol/L, and random blood sugar was 8.6 mmol/L. Considering the clinical presentation, we carried out varicella polymerase chain reaction (PCR) testing in CSF, which was positive.

Considering the clinical findings of a flaccid paralysis with a zoster rash with EMG evidence, a diagnosis of brachial plexopathy due to varicella infection was made. The patient was started on intravenous acyclovir and intravenous dexamethasone. Intravenous acyclovir was continued for 14 days, and on day 14, repeat CSF analysis was done, which showed negative varicella PCR. Gabapentin was prescribed for analgesia. The patient made a gradual recovery from weakness over the days with support from physiotherapy. She was reviewed in medical clinic 2 weeks from discharge, and complete neurological recovery was noted.

## Discussion

Meningoencephalitis and postherpetic neuralgia are the common neurological manifestations of varicella infection. Other infrequent but reported complications include cranial nerve palsies, peripheral motor neuropathy, myelitis, and Guillain–Barré syndrome [4].

Peripheral motor neuropathy is a very rare neurological sequela of VZV infection. Epidemiological studies show a motor involvement in 1–5% of patients with herpes zoster [7]. Pathophysiology of brachial neuritis due to VZV is yet to be elucidated, but clearly it is multifactorial. After primary infection, VZV becomes latent in the dorsal root ganglia. Reactivation of VZV is influenced by age, disease-related immunocompromise, and iatrogenic immunosuppression leading to appearance of rash and parathesia in a restricted dermatomal distribution. Spread of VZV from dorsal root ganglia to anterior root can explain the motor weakness.

Evidence from imaging, neurophysiological studies, and postmortem histopathological studies provides insight into the pathophysiological changes in brachial neuritis. Zubair *et al*. reported the largest series of patients with brachial neuritis due to VZV [8]. Increased T2 signal in the plexus, nerve enlargement, and denervation changes in the muscles innervated by the plexus are some specific magnetic resonance imaging (MRI) findings described in this study. T2 hyperintensity and contrast enhancement are described by Choi *et al*. for two patients [9, 10]. MRI may be very effective in precise localization of the lesion within the nervous system as the clinical findings will not be able to differentiate root, plexus, or more peripheral root lesion accurately. Neurophysiology studies demonstrate denervative pattern on EMG as in our patient, but precise localization is not possible with neurophysiology studies [11]. Fabian *et al*. reported the first case of zoster-associated limb paralysis in which histology of the brachial plexus showed extensive lymphocytic infiltration, myelin breakdown, and preservation of axons without vasculitis. Interestingly, anterior horn necrosis was not observed in the pathological specimen, although there was marked lymphocytic cuffing [12].

In the literature, very few cases of brachial plexopathy are described. A case of brachial plexopathy with severe manifestation of radial nerve palsy was reported by Jeevarethinam *et al*. [13]. Ohtake *et al*. [14] and Ismail *et al*. [15] described two cases of brachial plexopathy secondary to VZV infection. Bilateral diaphragmatic paralysis with brachial neuritis was described by Hoque *et al*. [16] following thoracic VZV infection. Postherpetic neuralgia is more commonly reported in zoster-associated plexopathy. A study by Zubair *et al*. reported 100% patients having neuralgia at 1 month after the rash [8]. The onset of motor involvement typically coincides with the development of pain and skin eruption [17]. But there are reports of delayed onset motor weakness following the VZV infection [18]. Our patient’s presentation is atypical as the motor weakness preceded the cutaneous eruption mimicking an acute stroke, but she had severe neuropathic pain of the limb at the onset.

Currently, there is no clear consensus on the management of zoster-associated plexopathy, and many treatment options are available without clear evidence on their effectiveness. Acyclovir is commonly used to treat patients with zoster-associated plexopathy, but there is no evidence that it hastens the recovery from paralysis [19]. Corticosteroids are used to treat zoster-associated paralysis [20–22]. Han *et al*. reported that corticosteroids given acutely during zoster infection are ineffective in preventing postherpetic neuralgia [20]. Whitley *et al*. in a randomized placebo-controlled trial concluded that, in localized herpes zoster, combined acyclovir and prednisone therapy can improve quality of life [23]. Direct steroid administration to the brachial plexus is used in few cases with success [24, 25]. Pulsed radiofrequency treatment or classic radiofrequency ablations are also tried at the experimental level in treatment of herpes zoster infections [26]. Sáenz-Farret *et al*. reported a case of zoster-associated plexopathy successfully treated with intravenous immunoglobulin [27]. Our patient was given intravenous acyclovir for 14 days and intravenous steroids for 72 hours, and we noticed a gradual improvement in motor function and pain. In addition, she received gabapentin for pain relief. The patient was started on a physiotherapy and rehabilitation program to prevent muscle atrophy and contractures.

## Conclusion

Brachial plexus neuritis due to varicella zoster infection should be considered in an acute monoparesis of a limb as it is a treatable and a reversible condition. Typical cutaneous eruptions may follow or precede the paresis, but severe pain involving the limb may give a clue to the possibility of the varicella zoster infection.

## Data Availability

Data sharing not applicable to this article as no datasets were generated or analyzed during the current study.
